# Self-care, resilience, and caregiver burden in relatives of patients with advanced cancer: results from the eQuiPe study

**DOI:** 10.1007/s00520-021-06365-9

**Published:** 2021-07-03

**Authors:** Janneke van Roij, Linda Brom, Dirkje Sommeijer, Lonneke van de Poll-Franse, Natasja Raijmakers, on behalf of the eQuiPe study group

**Affiliations:** 1 Research & Development, Netherlands Comprehensive Cancer Organization (IKNL), PO box 19079, 3501 DB Utrecht, The Netherlands; 2 CoRPS-Center of Research On Psychology in Somatic Diseases, Department of Medical and Clinical Psychology, Tilburg University, Tilburg, The Netherlands; 3 Netherlands Association for Palliative Care (PZNL), Utrecht, The Netherlands; 4 Libra Rehabilitation and Audiology, Tilburg, The Netherlands; 5 Department of Medical Oncology, Amsterdam University Medical Center, Amsterdam, The Netherlands; 6 Department of Medical Oncology, Almere, The Netherlands; 7 Division of Psychosocial Research and Epidemiology, Netherlands Cancer Institute, Amsterdam, The Netherlands

**Keywords:** Self-care, Resilience informal caregivers, Palliative care, Quality of life, Advanced cancer, Caregiver burden

## Abstract

**Purpose:**

Relatives are often involved in caregiving for patients with advanced cancer and carry a heavy burden. Self-care and resilience might be beneficial to enhance their wellbeing and burden-bearing capacity. This study assessed the engagement in self-care and resilience in relatives of patients with advanced cancer and its association with their caregiver burden.

**Methods:**

This study analyzed baseline data of the eQuiPe study, a prospective longitudinal, multicenter, observational study on quality of care and life of patients with advanced cancer and their relatives in which self-care (Self-care Practices Scale), resilience (Connor-Davidson Resilience Scale), and caregiver burden (Zarit Burden Interview (ZBI)) of relatives were included. Their scores were compared with a gender- and age-matched normative population. Multivariable logistic regression analysis was performed to assess the association between self-care and resilience with caregiver burden.

**Results:**

Most of the 746 relatives were the patient’s partner (78%) and 54% reported to be an informal caregiver of the patient. The median hours of caregiving a week for all relatives was 15 and 11% experienced high caregiver burden (ZBI > 20). Relatives who reported a high caregiver burden engaged less often in self-care (OR = .87) and were less resilient (OR = .76) compared to relatives with low/medium caregiver burden. Relatives with high caregiver burden were younger (OR = .96), highly educated (OR = 2.08), often reported to be an informal caregiver of the patient (OR = 2.24), and were less well informed about the importance of self-care (OR = .39).

**Conclusion:**

A significant number of relatives of patients with advanced cancer experienced high caregiver burden. As more self-care and resilience were associated with lower experienced caregiver burden, creating awareness of the beneficial potential of self-care is important. Future studies should illuminate the causal relation.

**Trial registration number:**

NTR6584 (date of registration: 30 June 2017)

## Background

The responsibility of caring for patients with a life-threatening illness such as advanced cancer is increasingly placed on their relatives [1]. As the number of patients living with advanced cancer is rising and their prognosis is improving [2], the number of relatives and other informal caregivers who are providing care and support to these patients is also likely to increase. Relatives of patients with advanced cancer often experience that caring for their loved one is fulfilling but may also carry a high caregiving burden. Informal caregivers of patients with advanced cancer typically provide 18 to 33 h a week care for their loved one [3, 4]. Furthermore, a systematic literature review on caregiver burden of informal caregivers of elderly patients with cancer showed that up to 35% of these caregivers experienced a high burden [5]. Moreover, informal caregivers of patients with advanced cancer often experience a low quality of life [3, 4, 6–10]. More specifically, for these informal caregivers, high rates of depression and anxiety are found [3, 4] as well as feelings of social isolation [9, 11] and loss of self-identity [10].

Caregiver burden has been defined as a “multidimensional biopsychosocial reaction resulting from an imbalance of care demands relative to caregivers’ personal time, social roles, physical and emotional states, financial resources, and formal care resources given the other multiple roles they fulfill” [12]. This definition suggests that the *balance* between burden and burden-bearing capacity in informal caregivers is crucial for their wellbeing and may prevent them from developing health issues themselves [13]. Moreover, higher caregiver burden in informal caregivers is associated with poorer physical and mental health of patients with advanced cancer [14]. When caregiving becomes a structural demand or its intensity increases, it is essential to restore the imbalance to prevent negative consequences in informal caregivers and patients. This imbalance can be restored by either decreasing the burden, for example, by respite care or by enhancement of the burden-bearing capacity of caregivers.

Resilience might contribute to burden-bearing capacity of informal caregivers, as it appears to be a predictor of adequate adaptation to negative life events [15]. Quantitative studies have shown that resilience in informal caregivers of patients with advanced cancer is related to less depression, better health, and positive social support and might be a protective factor for caregiver burden [16–18]. Another promising approach to enhance the burden-bearing capacity of informal caregivers seems promoting self-care. Self-care has been defined as a “process of purposeful engagement in practices that promote overall health and wellbeing of the self” [19]. Research on self-care in informal caregivers of patients with cancer is scarce. To our knowledge, only one study from Dionne-Odom et al. showed that low engagement in self-care practices was associated with more anxiety, depression, and lower mental quality of life in informal caregivers of patients with advanced cancer [4].

Overall, self-care and resilience may have potential to enhance the burden-bearing capacity of relatives of patients with advanced cancer and decrease their experienced caregiver burden. However, these concepts have received little attention yet. Therefore, this study aimed to assess the association between self-care engagement and resilience with perceived caregiver burden in relatives of patients with advanced cancer.

## Methods

### Study design

A prospective, longitudinal, multicentre, observational study on the experienced quality of care and quality of life of patients with advanced cancer and their relatives was conducted in the Netherlands (eQuiPe study). Patients were invited by their treating physician in the 40 participating hospitals or were self-enrolled between November 2017 and January 2020. Patients were contacted by phone by the research team to discuss participation and all patients were asked if a relative was interested in participating in the study. After giving written informed consent, patients and relatives completed a questionnaire every 3 months till the patient’s death. Questionnaires were completed on paper or online via the Patient Reported Outcomes Following Initial treatment and Long-term Evaluation of Survivorship (PROFILES) registry [20]. Clinical data of the patient was obtained by linking the information to the Netherlands Cancer Registry (NCR). The study was exempted from medical ethical review according to the Dutch Medical Research Involving Human Subjects Act (WMO), declared by the Medical Research Ethics Committee of the Antoni van Leeuwenhoek hospital (METC17.1491). The study is registered as NTR6584 in the Netherlands Trial Register. Details of the study protocol are reported elsewhere [21].

### Study population

Relatives of patients with advanced cancer (metastatic solid cancer stage IV) were eligible to participate. To limit inclusion of patients with a relatively long prognosis, additional inclusion criteria for breast and prostate cancer were respectively metastases in multiple organ systems and castration-resistant disease. Both patients and relatives had to be 18 years or older and be able to complete a Dutch questionnaire. In total, 1695 patients and 1171 relatives gave written informed consent. Of these relatives, 340 (29%) dropped out before baseline assessment due to various reasons (decreasing health or death of the patient (7%), too busy (1%), too confronting (1%), or unknown reason (19%)), resulting in 831 relatives (71%) who responded to the baseline questionnaire. For this study, we used baseline data of relatives in the eQuiPe study and randomly selected one relative per patient [39] patients had multiple relatives in the study to avoid dependent measures. This resulted in 746 relatives of unique patients with advanced cancer.

### Measures

#### Caregiver burden

Caregiver burden was measured by the 12-item Zarit Burden Interview (ZBI) [22] using a five-point Likert scale between “never” and “nearly always.” Total sum score ranges between 0 and 48, where higher scores indicate a greater caregiver burden. Cut-off scores of the ZBI are as follows: 0–10: low caregiver burden, 11–20: medium caregiver burden, and > 20: high caregiver burden. The ZBI has good psychometric properties and has been validated in informal caregivers of advanced cancer patients [22–24].

#### Self-care

Self-care was measured by the Personal Self-care subscale of the Self-care Practices Scale (SCPS) [25, 26]. The Personal Self-care subscale consists of nine items using a 5-point Likert scale ranging from “never” to “very often.” Respondents were asked to indicate how often they engage in self-care activities. The sum score ranged between 0 and 36, where higher scores indicate more self-care. Mean scores were calculated when all items of the Personal Self-Care scale were completed. The SCPS was originally developed for healthcare professionals; the psychometric properties of the self-care scale are good [25].

#### Resilience

Resilience, the extent to which people are able to “bounce-back” after negative life events and their adaptability, was measured with a short version of the validated Connor-Davidson Resilience Scale (CD-RISC 2) [27]. This short version included two items, using a 5-point Likert scale ranging from “not at all true” to “almost always true.” Mean scores were calculated when both items of the Resilience Scale were completed. A higher sum score (range 0–8) indicates more resilience [28]. The CD-RISC has been adequately validated in the general population and patients with psychiatric or medical conditions [29] but had not previously been used in caregivers of patients with advanced cancer.

#### Socio-demographics and clinical characteristics

Socio-demographic characteristics including gender, age, marital status, having children, educational level, and the nature of the relationship to the patient were all self-reported. To assess if relatives considered themselves as an informal caregiver, three self-developed questions were used: “Are you an informal caregivers of your relative with cancer?” (yes/no), “How many hours a week do you provide care?” (open-ended question), and “To what extent did a health care professional explain to you that it is also important to take care of yourself and not only of your relative?” The latter used a 5-point Likert scale ranging from “bad” to “perfect.” Clinical characteristics of the patients that were linked to the relative included primary tumor type, time since primary diagnosis (at time of patients’ baseline questionnaire completion), and comorbidities assessed with the Self-administered Comorbidity Questionnaire (SCQ) [30].

#### Normative population

Data of a normative population from 2018 were obtained from CentERpanel, an online household panel that is representative of the Dutch population [20]. Individuals from the normative population (n = 620) were matched (1:1) based on gender and age categories of relatives of patients with advanced cancer to compare their self-care and resilience scores.

### Statistical analysis

Descriptive statistics were performed to examine the sociodemographic characteristics, self-care, and resilience of relatives who experienced low, medium, or high caregiver burden. Because relatives often do not recognize their caregiving role or activities, we used the full sample of relatives regardless of their self-reported status (being an informal caregiver yes/no) in our analysis. Resilience and self-care scores of relatives with low, medium, or high caregiver burden were compared to a gender- and age-matched normative population by means of ANOVA analysis with Tukey post hoc tests. A chi-square test was conducted to compare subgroups based on the amount of experienced caregiver burden (low, medium, high) on being informed by health care professionals about the importance of self-care. Cronbach alpha showed that the reliability of the Personal Self-care scale (0.73 for relatives and 0.73 for normative population), resilience (0.73 for relatives and 0.69 for normative population), and caregivers’ burden (0.88 for relatives) was adequate. A logistic multivariable regression analysis was performed to examine the association between self-care and resilience levels (independent continuous variables) with caregiver burden (dependent categorical variable: high versus low/medium caregiver burden). Multiple imputation was applied prior to the logistic regression analysis to handle missing data which ranged between 0 and 10% per variable and were not missing completely at random. Multiple imputation did not affect the results of the analysis (when compared to the regression results based on the original data). The following covariates were included: age, educational level, the nature of the relation to the patient (e.g., being partner, a daughter/son, or other family or friend), considering oneself to be an informal caregiver of the patient, and being informed about the importance of self-care because univariate analyses showed that relatives with high caregiver burden differed significantly compared to relatives with low and medium caregiver burden (all *p* < 0.05). While gender was not significant (*p* = 0.31), gender was included based on previous studies showing relevant gender differences in informal caregivers of patients with advanced cancer [31–33]. A p-value of < 0.05 was considered statistically significant. All analyses were performed in STATA version 16.

## Results

### Socio-demographic characteristics and caregiver burden of relatives

Sixty percent of relatives were female with a mean age of 61 years (range 18–87) (Table 1). Most relatives were the patient’s partner (78%) and the median hours of caregiving a week was 15. The mean score of all relatives on caregiver burden was 10 (SD 7.3), indicating low caregiver burden. For those relatives reporting to be informal caregivers (54%), the mean score of caregiver burden was 11 (SD 7.5) and 14% of them experienced a high caregiver burden.

**Table 1:** Socio-demographic characteristics of relatives of patients with advanced cancer (n = 746)

	All relatives (n = 746)	Relatives with low caregiver burden (n = 420)^a^	Relatives with medium caregiver burden (n = 230)^a^	Relatives with high caregiver burden (n = 81)^a^	p-value
	*n* (%)	*n* (%)	*n* (%)	*n* (%)	
Gender					
Male	297 (40)	156 (37)	105 (46)	28 (35)	.07
Female	449 (60)	264 (63)	125 (54)	53 (65)	
Age					
Mean (SD), range	61 (13), 18–87	63 (12), 24–86	50 (13), 18–87	56 (14), 23–83	< .001*
18–54 years	178 (24)	81 (20)	60 (27)	34 (43)	
55–63 years	168 (23)	86 (21)	60 (27)	20 (25)	
64–69 years	181 (24)	115 (28)	49 (22)	13 (16)	
≥ 70 years	193 (26)	122 (30)	54 (24)	12 (15)	
Educational level					
Low	199 (27)	129 (31)	52 (23)	15 (19)	.01*
Medium	328 (44)	184 (44)	105 (46)	34 (42)	
High	212 (28)	101 (24)	73 (32)	32 (40)	
Relationship to patient					
Partner	583 (78)	325 (78)	186 (82)	60 (74)	.01*
Daughter/son	99 (13)	50 (12)	28 (12)	19 (23)	
Other family member or friend	58 (8)	41 (10)	14 (6)	2 (2)	
Marital status					
With partner	717 (96)	402 (96)	221 (96)	79 (98)	.79
No partner	28 (4)	17 (4)	9 (4)	2 (2)	
Having children					
Yes	609 (82)	348 (84)	176 (77)	71 (88)	.03*
No	132 (18)	68 (16)	54 (23)	10 (12)	
Being an informal caregiver of patient					
Yes	405 (54)	205 (49)	140 (61)	55 (68)	.001*
No	336 (45)	211 (51)	90 (39)	26 (32)	
Hours of caregiving per week^b^	n = 373	n = 189	n = 131	n = 49	.96
Median (25%, 75%)	15 (7, 28)	15 (5, 27)	15 (8, 29)	18 (9, 30)	
Missing	32 (8)	16 (8)	9 (6)	6 (1)	
Primary tumor of patient					
Lung	203 (27)	121 (28)	59 (26)	23 (28)	.07
Colorectal	121 (16)	83 (19)	29 (13)	9 (11)	
Breast	94 (13)	54 (12)	24 (10)	16 (20)	
Prostate	82 (11)	52 (12)	23 (10)	7 (9)	
Other	174 (24)	93 (22)	68 (30)	13 (16)	
Missing	71 (10)	31 (7)	26 (11)	13 (16)	
Time since primary diagnosis of patient					
< 1 year	225 (30)	133 (32)	73 (32)	15 (19)	.12
1–5 years	326 (44)	177 (42)	99 (43)	40 (49)	
> 5 years	124 (17)	80 (19)	31 (13)	13 (16)	
Missing	71 (10)	30 (7)	27 (12)	13 (16)	
#Physical comorbidities patient					
None	350 (47)	189 (45)	113 (49)	42 (52)	.58
1	234 (31)	139 (33)	70 (30)	20 (25)	
> 1	162 (22)	92 (22)	47 (20)	19 (23)	

Abbreviations: *SD* = standard deviation

Notes: Education levels are categorized according to International Standard Classification of Education guidelines

Missings did not exceed 5% unless stated otherwise

^a^Subpopulations based on caregiver burden do not add up to 100% (n = 746) due to missingness on the ZARIT Burden Interview (n = 15)

^b^Hours of caregiving is a conditional item and only reported by relatives who reported to be a caregiver of the patient (n = 405)

*p-value < .05 is considered significant

### Socio-demographic characteristics of relatives according to level of caregiver burden

Eleven percent of all relatives experienced a high caregiver burden, 31% experienced a medium caregiver burden, and 57% a low caregiver burden. Relatives with high caregiver burden were higher educated compared to relatives with low or medium caregiver burden (*p* < 0.01). They also were more often a child of the patient compared to being a partner or other family (*p* = 0.01). Moreover, relatives with a high caregiver burden more often reported to be an informal caregiver of the patient (68%), compared to relatives with a medium (61%) or low (49%) caregiver burden (*p* = 0.001). The average number of caregiving hours per week of these relatives did not differ between those with a low, medium, or high caregiving burden (*p* = 0.96).

### Self-care and resilience

Relatives with high caregiver burden were less resilient than the normative population (*p* < 0.001) while relatives with low caregiver burden were more resilient (*p* < 0.05) than the normative population (Table 2). All relatives, irrespective of their level of caregiver burden, were less likely to engage in self-care activities compared to the normative population (*p* < 0.001). Twenty-one percent of the relatives with low caregiver burden, 27% of the relatives with medium caregiver burden, and 44% of the relatives with high caregiver burden felt they had been poorly informed about the importance of self-care (*p* < 0.001).

**Table 2:** Self-care and resilience in relatives of patients with advanced cancer by level of caregiver burden (n = 746) and the normative population (n = 620)

	Relatives with low caregiver burden (n = 420)^a^	Relatives with medium caregiver burden (n = 230)^a^	Relatives with high caregiver burden (n = 81)^a^	Normative population (n = 620)
	Mean (SD)	Mean (SD)	Mean (SD)	Mean (SD)
Resilience (total score 0–8)	6.3 (1.6)*	5.9 (1.5)	5.3 (1.5)*	6.0 (1.4)
Self-care (total score 0–36)	21.0 (4.6)*	19.1 (4.4)*	17.3 (4.5)*	22.0 (4.5)
Items self-care measure (total score 0–4)				
I participate in physical activities	2.3 (1.1)*	2.3 (1.1)*	2.0 (1.0)*	2.6 (1.1)
I laugh	2.9 (.7)	2.5 (.7)*	2.2 (.8)*	3.0 (.7)
I am involved in spiritual activities	.7 (1.1)*	.8 (1.1)	.8 (1.1)	1.0 (1.1)
I get enough sleep for my body	2.8 (.8)	2.4 (.9)*	2.1 (.9)*	2.9 (.8)
I spend time with people I care about	3.2 (.6)	2.9 (.7)*	2.7 (.8)*	3.1 (.7)
I participate in activities that I enjoy	2.7 (.8)	2.4 (.9)*	2.1 (.9)*	2.8 (.8)
I accept help from others	2.2 (.8)	2.0 (.8)*	2.1 (.8)	2.3 (.8)
I experience physical intimacy	2.2 (1.1)	2.0 (1.0)	1.7 (.9)*	2.1 (1.1)
I do things to fulfill my emotional needs	2.0 (1.1)*	1.9 (.9)*	1.7 (.9)*	2.3 (.9)
	*n (%)*	*n (%)*	*n (%)*	*p-value*^*b*^
Informed about the importance of self-care				
Bad	90 (21)	61 (27)	36 (44)	< .001*
Reasonable	74 (18)	65 (28)	22 (27)	
(Very) good/perfect	200 (48)	89 (39)	21 (26)	
Missing	56 (13)	15 (7)	2 (2)	

Abbreviations: *SD* = standard deviation

^a^ANOVA tests with Tukey post hoc tests were conducted to compare subgroups based on the amount of experienced caregiver burden (low, medium, high) with the normative population

^b^A chi-square test was conducted to compare subgroups based on the amount of experienced caregiver burden (low, medium, high) on being informed by health care professionals about the importance of self-care

^*^p-value < .05 is considered significant

### Associations between self-care, resilience, and caregiver burden

Relatives with high caregiver burden engaged less often in self-care activities (OR = 0.87, 95%CI 0.82–0.92) and were less resilient (OR = 0.76, 95%CI 0.65–0.89) compared to relatives with low or medium caregiver burden (Table 3). Also, relatives who experience a high caregiver burden were less often well informed about the importance of self-care by health care professionals (OR = 0.39, 95%CI 0.21–0.73). Being younger (OR = 0.96, 95%CI 0.94–0.99), highly educated (OR = 2.08, 95%CI 1.00–4.32), and being an informal caregiver of the patient (OR = 2.24, 95%CI 1.28–3.93) were positively associated with high caregiver burden.

**Table 3:** Odds ratios of the multivariable logistic regression model estimating the associations of self-care and resilience *with high caregiver burden* in relatives of patients with advanced cancer (n = 746)

	Odds ratio (95%CI)	p-value
Gender		
Male	1	
Female	1.58 (.92–2.71)	.08
Age	.96 (.94–.99)	.004*
Education		
Low	1	
Medium	1.52 (.77–3.00)	.23
High	2.08 (1.00–4.32	.05
Relation to patient		
Partner	1	
Daughter/son	1.07 (.46–2.51)	.87
Other family or friend	.40 (.09–1.82)	.24
Informal caregiver		
No	1	
Yes	2.24 (1.28–3.93)	.01*
Informed about self-care		
Bad	1	
Reasonable	.63 (.34–1.19)	.16
(Very) good/perfect	.39 (.21–.73)	.003*
Resilience	.76 (.65–.89)	.001*
Self-care	.87 (.82–.92)	< .001*

Abbreviations: *CI* = confidence interval

Notes: -2LL = -210.93, adjusted R^2^ = .11

^*^p-value < .05 is considered significant

## Discussion

This study shows that a significant part of relatives of patients with advanced cancer experience a high caregiver burden. Relatives who experience a high caregiver burden engage less often in self-care activities and are less resilient than the general population and compared to relatives who experience a lower caregiver burden. Moreover, relatives with high caregiver burden are also younger, higher educated, more often define themselves as being an informal caregiver of the patient, and are less often well informed about the importance of self-care compared to relatives with a lower caregiver burden.

Some findings deserve attention. Overall, 11% of the informal caregivers of patients with advanced cancer experience a high caregiver burden. This percentage is similar to findings of other studies among informal caregivers of patients with advanced cancer in the UK and Thailand [34–37]. However, a systematic literature review regarding the prevalence of caregiver burden in relatives of *elderly* cancer patients showed that high burden ranged from 1% to greater than 35% [5].

We also found that relatives of patients with advanced cancer engage less often in self-care activities compared to the general population. It is clearly challenging to engage in self-care activities when time and energy are limited due to caregiving activities. Especially relatives with high caregiver burden engage little in self-care activities which may result in a lower wellbeing and in an imbalance in burden and burden-bearing capacity in informal caregivers. Stenberg et al. found that informal caregivers of patients with cancer often restrict their leisure time and social time to meet the patients’ needs [38] Some informal caregivers also tend to give priority to the patients’ needs over their own [39]. Clearly, when the patient’s needs increase over time due to disease progression, relatives will even have less time available for self-care activities. This is worrisome as self-care activities are important for the wellbeing of relatives and for their ability to continue caregiving activities. Less self-care in caregivers has been found to be associated with poorer performance in caregiving activities, such as being less prepared for caregiving tasks and responsibilities [4]. Hence, self-care in relatives is important for the wellbeing of the relative and may also be beneficial for the patient.

Self-care for relatives who care for a patient with advanced cancer is important and this can be emphasized by healthcare professionals, especially in palliative care where quality of life of both patients and relatives is an important focus of care [40]. Unfortunately, our study showed that a significant part of the relatives reported to be poorly informed about the importance of self-care. To our knowledge, no other studies regarding the information about self-care for relatives in the advanced cancer setting are present.

Clearly, the quality of life of relatives of patients with advanced cancer is affected and decreases further as the disease progresses [6, 41]. Early palliative care [42, 43] including caregivers support such as respite care might be potential interventions to improve the quality of life of relatives. Unfortunately, the support for these relatives seems no common practice, as unmet health care needs are still prevalent in this population [44]. A barrier for receiving adequate support as mentioned by informal caregivers was the focus of care on the patient, rather than on the relatives [45]. According to a previous qualitative study among informal caregivers of patients with cancer [46], health care professionals can support informal caregivers by establishing a personal relation. Being seen and heard by health care professionals may enhance resilience in informal caregivers [46]. Other factors that may foster resilience that were mentioned are as follows: the availability of palliative care; adequate information and communication on illness, prognosis, and death, and facilitating a good relationship between the informal caregiver and the patient [46]. These factors may also be associated with caregiver mastery, the caregivers’ sense of control over their situation [47]. Caregiver mastery, but also how patients and relatives cope with their situation, may influence the wellbeing and burden as experienced by relatives [7, 48].

Last, we found no association between caregiver burden and type of relationship (e.g., partner, child, or other family or friend). This was unexpected as a recent review on the risk factors of caregiver burden showed that living in the same household was a risk factor, together with being female, low educational level, higher number of hours spent caregiving, and lack of choice in being a caregiver [49]. Another study showed that especially adult daughters of patients with cancer experience high levels of caregiver burden [50]. A possible explanation for these differences might be that we included relatives of patients with advanced cancer, while Adelman et al. [49] included informal caregivers of patients with various illnesses with a more chronic (longer term) character, including stroke. For these relatives, the caregiver burden will persist longer and is more unpredictable due to the possible cognitive or behavioral changes in patients, compared to the often shorter and more predictable illness trajectory of advanced cancer [51].

We also found that younger age is associated with higher caregiver burden; this is in line with a previous study among family caregivers of elderly patients with cancer [5]. Younger caregiver may experience more burden because their caregiving interferes with their personal and social activities [52]. The social activities and network of both the patient and the relative are often more extensive when younger, which might be beneficial (more support and resources) but also burdensome (more to juggle).

### Limitations

This study has some limitations. First, it is unclear to what extent relatives were engaged in self-care activities and were resilient before the cancer diagnosis of the patient. It is possible that our study population differed from the normative population prior to the cancer diagnosis of the patient as we only matched on age and gender. Second, relatives might have interpreted the self-constructed questions regarding being an informal caregiver and the hours spent on caregiver differently, as we did not define informal caregiver and caregiving activities in the questionnaire. Some relatives may not consider themselves to be an informal caregiver while other relatives, who engaged in similar caregiving activities, did consider themselves to be an informal caregiver of the patient. Moreover, it is likely that the time spent on caring for the patient and also the caregiver burden is higher in relatives of patients who experience more symptoms or with disease progression [53]. Unfortunately, we did not assess whether the burden was higher for relatives of patient with more symptom burden or disease progression. Fourth, the Personal Self-Care Measure was initially developed and validated for social workers [25] and not validated in relatives of patients with advanced cancer. To our knowledge, no measurement instruments assessing self-care in relatives of patients with advanced cancer exist.

Last, this cross-sectional analysis only provides insight into associations, not in causal relations. Therefore, it is unclear if relatives with high caregiver burden experience less time to engage in self-care activities or if a lack of self-care activities leads to high caregiver burden.

### Practical implications and future research

It is important for health care professionals to be aware that younger and highly educated relatives of patients with advanced cancer are more at risk to high caregiver burden. Moreover, it is important to assess whether these relatives are resilient and engage in self-care activities because it can potentially protect them from high caregiver burden. Also, as harmful effects of self-care are unlikely, it is an appropriate step to inform relatives about the importance of their wellbeing and the role of self-care. More research is needed to find ways to increase caregiver wellbeing and their burden-bearing capacities, such as caregiver support, self-care, and resilience, and to clarify directional effects by means of longitudinal research. Also, the relation between the two concepts (self-care and resilience) needs to be further explored as more resilient relatives may also be more prone to engage in self-care activities and vice versa. To adequately assess these concepts, the validation of appropriate measures for relatives is needed.

## Conclusions

A significant number of relatives of patients with advanced cancer experience high caregiver burden. More self-care and resilience are associated with lower caregiver burden, but relatives’ engagement in self-care activities is still limited. Creating awareness of the potential of self-care could be beneficial for relatives, although more insight into the causal relation is needed. Future studies should focus on the potential of self-care to promote caregivers’ wellbeing and to enhance burden-bearing capacity of relatives of patients with advanced cancer.

## Data Availability

Since 2011, PROFILES registry data is freely available according to the FAIR (Findable, Accessible, Interoperable, Reusable) data principles for non-commercial (international) scientific research, subject only to privacy and confidentiality restrictions. The datasets analyzed during the current study are available through Questacy (DDI 3.x XML) and can be accessed by our website (www.profilesregistry.nl). In order to arrange optimal long-term data warehousing and dissemination, we follow the quality guidelines that are formulated in the “Data Seal of Approval” (www.datasealofapproval.org) document, developed by Data Archiving and Networked Services (DANS). The data reported in this manuscript will be made available when the eQuiPe study is completed.
